# Contingent capture of involuntary visual attention interferes with detection of auditory stimuli

**DOI:** 10.3389/fpsyg.2014.00528

**Published:** 2014-06-02

**Authors:** Marc R. Kamke, Jill Harris

**Affiliations:** The Queensland Brain Institute, The University of QueenslandSt. Lucia, QLD, Australia

**Keywords:** selective attention, contingent capture, spatial attention, bottleneck, visual, auditory

## Abstract

The involuntary capture of attention by salient visual stimuli can be influenced by the behavioral goals of an observer. For example, when searching for a target item, irrelevant items that possess the target-defining characteristic capture attention more strongly than items not possessing that feature. Such *contingent capture* involves a shift of spatial attention toward the item with the target-defining characteristic. It is not clear, however, if the associated decrements in performance for detecting the target item are entirely due to involuntary orienting of spatial attention. To investigate whether contingent capture also involves a non-spatial interference, adult observers were presented with streams of visual and auditory stimuli and were tasked with simultaneously monitoring for targets in each modality. Visual and auditory targets could be preceded by a lateralized visual distractor that either did, or did not, possess the target-defining feature (a specific color). In agreement with the contingent capture hypothesis, target-colored distractors interfered with visual detection performance (response time and accuracy) more than distractors that did not possess the target color. Importantly, the same pattern of results was obtained for the auditory task: visual target-colored distractors interfered with sound detection. The decrement in auditory performance following a target-colored distractor suggests that contingent capture involves a source of processing interference in addition to that caused by a spatial shift of attention. Specifically, we argue that distractors possessing the target-defining characteristic enter a capacity-limited, serial stage of neural processing, which delays detection of subsequently presented stimuli regardless of the sensory modality.

## INTRODUCTION

It is commonly held that the human brain is equipped with selection mechanisms that filter and prioritize incoming sensory signals. Known as selective attention, these mechanisms can be captured *involuntarily* by highly salient events in the environment, or directed *voluntarily* according to goals or task demands (for review, see Yantis, 2000). The *involuntary* capture of visual attention, however, can also be influenced by the goals of an observer. Specifically, when attention is set to select an item with a particular characteristic, such as a specific color, irrelevant items possessing that property will involuntarily capture attention to a greater degree than items not sharing the feature (such as different colored or moving items; see Folk et al., 1992, 1994). For example, waiting at an intersection for a green signal, a driver may be seen to lurch forward following the appearance of a green turning arrow. Despite appearing in the wrong spatial location and being the wrong shape, the arrow captures the driver’s attention due to its (task-relevant) color (Ghorashi et al., 2003). Converging evidence suggests that such *contingent capture* involves a shift of spatial attention toward the item containing the target-defining feature, but it is not clear if the associated decrements in performance for detecting a target item are entirely due to involuntary orienting of spatial attention. Here, we address this question by determining whether the detection of auditory events is hindered during contingent capture of visual spatial attention.

There is now a wealth of evidence suggesting that involuntary contingent capture involves a shift in visuospatial attention. For example, behavioral studies have shown that irrelevant distractors that share the target’s defining characteristic spatially cue targets (Folk et al., 1992, 1994; Bacon and Egeth, 1994; Gibson and Kelsey, 1998) and improve identification when accompanied by a perceptual prime for the target (Folk et al., 2002). Importantly, equally salient distractors that do not share the target’s defining characteristic do not cause such cueing or priming effects. Using functional magnetic resonance imaging (fMRI) it has been shown that, compared to non-target colored distractors, target-colored distractors are associated with increased neural activity in parietal brain regions putatively involved in spatial attention (Serences et al., 2005). Similarly, electroencephalography studies have shown that contingent capture is associated with an enhanced N2pc event-related potential, which is believed to index a shift in visuospatial attention (e.g., Eimer and Kiss, 2008; Kiss et al., 2008; Leblanc et al., 2008; Lien et al., 2008; Ansorge et al., 2009, 2011; Brisson et al., 2009). In one compelling demonstration, targets were always presented within a central stream of stimuli whilst distractors were presented to the side of the central stream (Leblanc et al., 2008). Despite the fact that distractors were irrelevant to the task and never appeared at the target location, target-colored, but not non-target-colored distractors were associated with a decrement in behavioral performance and induced a shift in visuospatial attention, as indexed by the N2pc (for a similar result, see Jolicoeur et al., 2006).

Although there is strong evidence showing that the involuntary capture of visual spatial attention is contingent upon the behavioral goals of the observer (but see, e.g., Theeuwes, 2010), it is not clear if the associated decrements in behavioral performance are entirely due to orienting of spatial attention. Evidence for an additional interference is gained from studies showing that irrelevant distractors possessing the target-defining feature interfere with target detection even when shifts in visuospatial attention were prevented by presenting distractors at fixation (e.g., Ghorashi et al., 2003; Folk et al., 2008). To explain these effects Ghorashi and colleagues proposed a two-stage model in which stimuli must first pass an input filter that is tuned to the target’s defining feature before gaining access to a capacity-limited stage of processing that is serial in nature. Importantly, access to this second stage is unavailable while a distractor is being processed, thereby interfering with processing of the target itself.

If capacity-limited central resources add to the behavioral decrements observed with contingent capture, then processing of an irrelevant distractor item should interfere not only with detection of the associated target, but also with other tasks requiring central resources. Preliminary support for this hypothesis can be found in a study in which a concurrent auditory discrimination task was used to investigate the influence of cognitive load on contingent capture (Brisson et al., 2009). In that study, N2pc data revealed that the capture of visuospatial attention by target-colored distractors was reduced when participants undertook a demanding auditory task (Brisson et al., 2009). Interestingly, performance on the auditory task also varied across the distractor conditions, being poorest for target-colored items. The difference was, however, very small (albeit statistically significant; 83–86% across all conditions) and complicated by a speed-accuracy trade off. Prompted by this incidental observation, instead of asking if a secondary task influences contingent capture we investigated whether contingent capture influences cognitive processing of a secondary task. We hypothesized that if visual items that capture spatial attention enter a capacity-limited, serial stage of neural processing, then behavioral performance will be negatively impacted for both visual and auditory targets that appear while the distractor is being processed. We tested this hypothesis by investigating the influence of irrelevant visual distractors on visual and auditory target detecting. Distractors included an item that shared its color with the to-be-detected visual target as well as a non-target-colored item. To provide an additional test of the contingent capture effects a third distractor, which rotated but did not change color, was included.

## MATERIALS AND METHODS

### PARTICIPANTS

Twenty-four adult volunteers participated in the study (median age 32 years; range 23–53 years; 16 female). All participants had normal or corrected-to-normal vision and no known hearing problems. Study procedures were approved by the Medical Research Ethics Committee at The University of Queensland and fully informed, written consent was obtained from all participants.

### STIMULI

**FIGURE 1:**
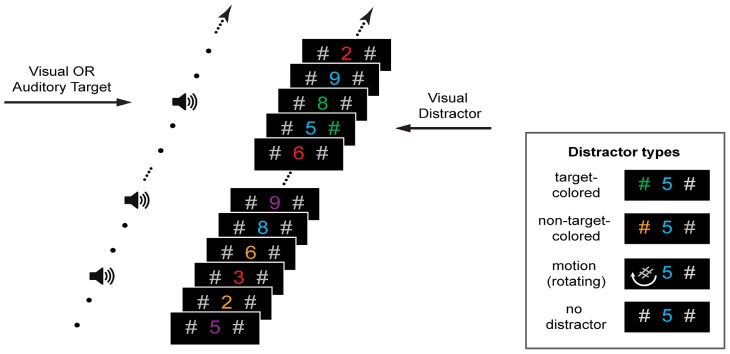
**Stimuli and trial design**. Participants fixated a central stream of rapidly presented digits and listened to tones. Their task was to detect either a number presented in a pre-defined color or a change in the tonal frequency. Prior to the presentation of a visual or auditory target (50% probability) either no distractor or one of three visual distractors was presented on the left or right of fixation. Distractors matched the color of the visual target, were in a different color, or did not change color but rotated (see insert).

Visual stimuli consisted of six digits of similar luminance (2, 3, 5, 6, 8, and 9; all within 1 cd/m^2^; ColorCAL colorimeter, Cambridge Research Systems) that were colored blue (RGB: 0, 0, 225), green (0, 115, 0), purple (140, 0, 170), red (213, 0, 0), or ochre (149, 79, 0). As shown in **Figure 1**, the digits were presented in a rapid stream at the center of a computer screen and were flanked 2° to the left and right by a gray (85, 85, 85) hash symbol. All stimuli were presented in Arial font on a black background and subtended 1.3° in height. Each stimulus was presented for 117 ms with a stimulus onset asynchrony of 150 ms. Visual targets were the number “3” or “8” presented in either blue, green, purple or red. Each participant was assigned a single target color for the duration of the experiment, but colors were counterbalanced across participants. A visual target was presented on 50% of trials and occurred randomly at position 9, 12, or 15 in the stream. Targets were always followed by nine non-target digits. Non-target digits were randomly generated from all numbers used (including “3” and “8”) and all non-target colors.

Auditory stimuli were presented free field at ~70 dB SPL (measured at the ear; Bruel and Kjaer, 2205) using desktop speakers placed directly to either side of the display screen. The “standard” auditory stimulus was a 1000 Hz pure tone with 70 ms duration, which was presented simultaneously with every third digit in the visual stream (see **Figure 1**). Auditory targets consisted of one of two “oddball” tones, one lower and one higher in frequency than the standard tone. To provide a perceptually equivalent difference between higher and lower oddballs the target tones differed from the standard by the same frequency ratio. Furthermore, to ensure that the difficulty of the auditory discrimination task was similar across participants, different high and low oddball tones were used depending on accuracy in a titration task (see Procedure). Targets had the following frequency ratio relative to the 1000 Hz standard tone: 1.4 (a high tone of 1400 Hz and a low tone 714 Hz), 1.2 (1200 and 833 Hz), 1.1 (1100 and 909 Hz), 1.05 (1050 and 952 Hz), 1.025 (1025 and 976 Hz), and 1.01 (1010 and 990 Hz). Thus, the ratio 1.01 was the most difficult to discriminate and the ratio 1.4 was the easiest. An auditory target was presented on the 50% of trials in which there was no visual target. Auditory targets were accompanied by both lateral hash marks, but no central digit. The central digit was not concurrently presented with an auditory target so that the task could be adapted for an evoked potential study in future research. Importantly, the central digit was absent whenever an auditory target was presented (i.e., following the no-distractor and all distractor conditions), so any differences in behavioral performance across the visual distractor conditions cannot be attributed to this factor.

On each trial, immediately prior to the appearance of a visual or auditory target one of either three visual distractors, or no distractor, was presented (in equal proportions). As shown in **Figure 1** (insert), distractors consisted of either one of the hash marks changing to the target color (target-colored distractor), one of the hash marks changing to a non-target color (non-target-colored distractor), or one of the hash marks rotating 540° over the display period (motion distractor). Distractors were equally likely to appear on the left and right sides of the central stream. On 25% of trials only the gray hash marks were presented (“no-distractor” control condition). Stimulus presentation was controlled using Presentation software (v14, Neurobehavioral Systems) running under Windows Vista on a laptop computer with a 17" LCD display (70 cm viewing distance) and a refresh rate of 60 Hz.

### PROCEDURE

Prior to the start of the experiment participants were given practice trials of the visual task alone (no auditory stimuli presented), the auditory task alone and then the full task. To ensure that performance on the auditory task was comparable between participants, and to avoid floor and ceiling effects, an auditory titration task preceded the practice trials. The titration task consisted of eight high and eight low oddball tones from each of the six ratio levels (see Stimuli), presented randomly in two blocks. The oddball pair used in the experiment was the most difficult to discriminate in which the participants’ accuracy was at least 80%. Nine participants completed the most difficult discrimination (1.01 ratio), 10 the 1.025 ratio, 1 the 1.1 ratio, and 2 participants each completed the 2 easiest oddball pairs (1.2 and 1.4 ratio).

The experiment proper consisted of six blocks of 48 trials; 36 trials for each of the two target (visual or auditory) and four distractor conditions. Experimental trials commenced with a fixation cross followed by the concurrent presentation of the visual and auditory streams (see **Figure 1**). The participant was tasked with detecting, as quickly and accurately as possible, the number presented in a pre-defined target color or a change in tonal frequency. A four-alternative forced choice response was made via key press on a standard keyboard, which was labeled “3” or “8” for visual targets and “LOW” or “HI” for auditory targets. For half of the participants in each group, the visual response buttons were the D (“3”) and F (“8”) keys, pressed using the left hand, while the auditory response buttons were the J (“LOW”) and K (“HI”) keys, pressed using the right hand. The remaining participants in each group used the J (“3”) and K (“8”) keys for response to visual stimuli, and the D (“LOW”) and F (“HI”) keys for auditory responses. The four targets were presented with equal probability. Feedback on accuracy was presented after each trial. Within each condition, trial length and distracter position (right or left) were counterbalanced. Self-paced rest breaks were offered between blocks.

### STATISTICAL ANALYSIS

Reaction times greater than 2.5 standard deviations from the mean for each condition, or that were less than 100 ms, were removed prior to analysis. This screening procedure eliminated less than 2.2% of the data (range across participants: 0.4–3.7%). Responses within each modality were then collapsed across the two target types (i.e., “3” or “8” for visual and “Low” or “High” for auditory). Differences in baseline behavioral performance between the visual and auditory tasks were investigated by comparing reaction times and accuracy in the no-distractor condition using two-tailed t-tests. To investigate contingent capture effects, individual responses for each distractor condition were expressed relative to the no-distractor baseline condition (i.e., visual distractor condition minus no-distractor condition). These difference data were then compared using repeated measures ANOVA with the factors distractor (target-colored, non-target-colored, motion) and Task (visual, auditory). Significant main effects were followed-up using planned comparisons between the three distractor conditions using Bonferroni adjusted, two-tailed *t*-tests (corrected alpha level = 0.017). Because reaction time data were generally moderately positively skewed and accuracy data negatively skewed, but to a smaller extent, data were normalized using a Log10 and square root (with reflection) transformation, respectively. The results of inferential analysis are based on this normalized data, but descriptive statistics are reported using the untransformed values. These transformations did not change the significance of any of inferential analyses. Statistical analyses were carried out using SPSS (v19; IBM).

## RESULTS

Mean reaction times in the control (no distractor) condition were faster for visual (632 ± 17 ms; *M* ± SE) compared to auditory (854 ± 59 ms) targets (mean difference = -0.1157, 95% CI = -0.1647 to -0.0667; *t*_2__3_ = -4.886, *p* < 0.001). Accuracy in the no-distractor condition was high and did not vary between the visual (96.3 ± 0.9%) and auditory (93.3 ± 1.9%) tasks (mean difference = -0.3759, 95% CI = -1.1092 to 0.3575; *t*_23_ = -1.060, *p* > 0.29).

**FIGURE 2:**
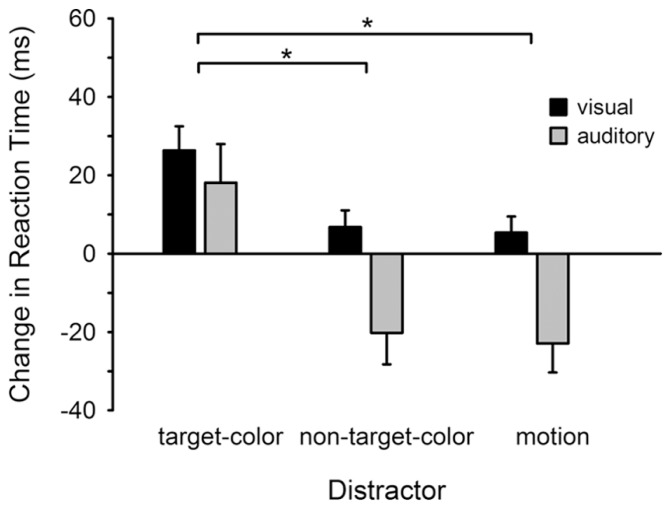
**Change in reaction time for detecting visual and auditory targets**. Reaction times are plotted for each distractor condition relative to the baseline (no-distractor) condition. Reaction times were delayed following the presentation of a target-colored distractor for both the visual and auditory tasks. *p < 0.05 (corrected for multiple comparisons). Error bars show within subjects (normalized) SEM (Cousineau, 2005).

**Figure 2** illustrates the influence of visual distractors on reaction times for both the visual and auditory tasks, relative to the no-distractor condition. For the visual task non-target-colored and motion distractors had little influence on reaction times, whereas the same distractors were associated with a substantial speeding of auditory responses (negative change in response time). Critically, for both tasks the slowest responses occurred in the target-colored distractor condition. Repeated measures ANOVA confirmed that reaction times varied across the distractor conditions (Distractor: *F*_2,4__6_ = 7.353, *p* = 0.002; $\eta_{p}^{2}$ = 0.242), but not between the visual and auditory tasks (Task: *F*_1,23_ = 2.330, *p* > 0.14; Distractor × Task: *F*_2,46_ = 1.135, *p* > 0.32). Specifically, reaction times were longer following the presentation of a target-colored distractor compared to both the non-target-colored (*t*_2__3_ = 2.875, *p* = 0.009) and motion (*t*_23_ = 3.113, *p* = 0.005) distractors. There was no difference between the non-target-color and motion conditions (*t*_23_ = 0.546, *p* > 0.58).

**FIGURE 3:**
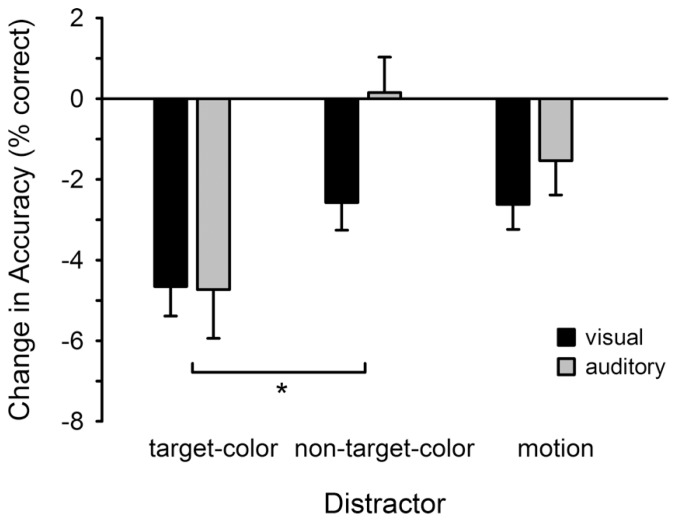
**Change in accuracy for detecting visual and auditory targets**. Accuracy is plotted for each distractor condition relative to the baseline (no-distractor) condition. Changes in accuracy were identical for the visual and auditory tasks. *p < 0.05 (corrected for multiple comparisons). Error bars show within subjects SEM.

**Figure 3** illustrates changes in accuracy for detecting visual and auditory targets, relative to the no-distractor condition. In general, accuracy was reduced in the presence of a visual distractor, with the highest reduction in the target-colored distractor condition. A repeated measures ANOVA confirmed that the change in accuracy varied across the distractor conditions (Distractor: *F*_2,46_ = 4.568, *p* = 0.016; $\eta_{p}^{2}$ = 0.166), but that there was no difference between the visual and auditory tasks (Task: *F*_1,23_ = 1.086, *p* > 0.30; Distractor × Task: *F*_2,46_ = 0.327, *p* > 0.72). Follow-up comparisons revealed that accuracy was lower in the target-colored compared to non-target-colored (*t*_23_ = 2.740, *p* = 0.012), but not motion (*t*_23_ = 1.964, *p* = 0.062), condition. There was no difference between the non-target-color and motion conditions (*t*_23_ = -1.038, *p* > 0.30).

## DISCUSSION

There is now a wealth of evidence showing that the extent to which visual events involuntarily capture attention depends on the goals (attentional set) of an observer. In the present study participants simultaneously monitored streams of visual and auditory stimuli for target items (digits of a particular color and atypical tones, respectively) while irrelevant visual distractors were presented to the left or right of fixation. Behavioral performance in the visual detection task was negatively impacted by distractors that possessed the target-defining characteristic compared to other distractors. Thus, the present results are consistent with the contingent capture hypothesis, which posits that involuntary capture of attention is influenced by attentional set (Folk et al., 1992, 1994). The novel observation is that the presentation of a visual distractor that possessed a target-defining feature also induced deficits in auditory target detection. Specifically, auditory performance was poorer following the presentation of a target-colored visual distractor compared to presentation of non-target colored and motion distractors. These results show that visual contingent capture affects processing not only of visual, but also auditory stimuli.

In the present study response times for detecting visual and auditory targets were significantly increased in the presence of a target-colored distractor compared to both non-target colored and motion distractors. The primary measure of interest was response time, as accuracy in the four-alternative forced-choice detection task was expected to be high. Nonetheless, for both the visual and auditory tasks the target-colored distractor was also associated with a reduction in accuracy compared to the non-target-colored condition, while there was no change in accuracy compared to the motion condition. This pattern of results suggests that the increase in reaction time following the presentation of a target-colored distractor, relative to the other distractors, was not merely due to a speed-accuracy trade off. Importantly, the results also cannot be attributed to differences across the distractor conditions in salience at the sensory level, as target and non-target colors were of equal luminance and, across participants, were the same colors.

In a previous study using a paradigm similar to that employed here it was shown that the target-colored distractor induced a shift of visuospatial attention to the distractor location, as indexed by the N2pc event-related potential (Leblanc et al., 2008). The present results suggest that, as well as capturing visuospatial attention, irrelevant visual stimuli that possess the characteristic feature of a to-be-detected visual target can interfere with auditory processing. It is well established that visual stimuli appearing at a certain location can enhance detection of auditory stimuli appearing at that location and hamper detection of sounds presented elsewhere (for review, see Spence and McDonald, 2004). Such effects of cross-modal spatial attention, however, are unlikely to account for the current results as the auditory stimulus was not spatially distinct. Indeed, the visual distractor was located only 2° from fixation, so any accompanying shift in visuospatial attention occurred within the bilateral auditory sources and below the spatial resolution of cross-modal cueing effects (e.g., Focker et al., 2010). Thus, our observed decrement in auditory performance following a target-colored distractor suggests that contingent capture involves a source of processing interference in addition to that caused by a spatial shift of attention.

It has been suggested that as well as causing a shift in visuospatial attention, contingent capture may induce a non-spatial capture of attention (Ghorashi et al., 2003). Initial support for this idea was garnered from the attentional blink phenomenon, which describes the decrement in detection of a second target (T2) when it appears soon after a first (T1; Ghorashi et al., 2003; Folk et al., 2008). In particular, it was shown that even when there was no requirement to report T1, detection of T2 was impaired if T1 shared critical features with the second target (Chun, 1997). Although the exact mechanisms have yet to be elucidated, it is commonly held that the attentional blink reveals a central bottleneck in neural processing (e.g., Raymond et al., 1992). Specifically, when T1 is detected it enters a capacity-limited, serial stage of processing that prevents or delays processing of T2 (for recent reviews, see Dux and Marois, 2009; Martens and Wyble, 2010). Further evidence supporting the idea that contingent capture may also involve a central bottleneck is provided by the observation that the N2pc component elicited by target-colored distractors was reduced when participants undertook a concurrent auditory task, which taxed central resources (Brisson et al., 2009). Moreover, it was recently reported that contingent capture is reduced or eliminated during the attentional blink (Du et al., 2013), suggesting that both phenomena depend on capacity-limited central resources. The notion that contingent capture involves such central resources can also account for the impairment we observed in auditory detection following the target-colored distractor.

Although we did not find any difference in the pattern of contingent capture effects between the visual and auditory detection tasks, data presented in **Figure 2** show that the presence of non-target-colored and motion distractors was associated with faster responses to auditory targets relative to the no-distractor condition. One explanation for this result is that the visual distractors may have acted as an alerting cue, as they were temporally predictive of a target. Critically, even if distractors had an alerting value this effect was identical across the distractor conditions and therefore cannot explain the selective slowing of responses to auditory targets associated with target-colored distractors. Moreover, it has been shown that eliminating the predictiveness of a distractor display that was similar to the one used here did not alter visual contingent-capture or the N2pc (Experiment 3, Leblanc et al., 2008). Nonetheless, if distractors did carry an alerting value then it could be argued that any cueing effect should manifest for all distractors in both the visual and auditory detection tasks. In this context it is possible that non-target-colored and motion distractors did facilitate responses to the auditory and visual targets, but for the latter any facilitation was counteracted by an involuntary shift of visuospatial attention toward the distractor item (Hickey et al., 2006). On this argument target-colored distractors presumably also cued (speeded) detection, however, that item would have gained access to a capacity-limited, serial stage of processing that interfered with identification of both visual and auditory targets.

Another interesting observation in the present study is that the absolute cost in response time following the presentation of a target-colored distractor, relative to the non-target-colored distractors, was numerically twice as large in the auditory task as it was in the visual task (see **Figure 2**). A similar result was reported previously when participants were required to maintain two attentional sets for color (such as for green and orange). In that study identification of a (e.g., orange colored) target was poorest following presentation of a distractor that matched the other attentional set (green; Moore and Weissman, 2010). The authors proposed that contingent capture involves not only a reduction in accuracy due to capacity-limited resources processing the distractor, but also an enhancement due to the attentional set entering the focus of attention (Moore and Weissman, 2011). Thus, when two attention sets must be maintained a target-colored distractor can interfere with detection not only by occupying central processing resources but also by impairing the ability to attend to a subsequent item whose color matches a different attentional set (Moore and Weissman, 2010). In line with this argument it is possible that the target-colored distractor in the present study captured visuospatial attention, allowing that item to enter a capacity-limited stage of processing that interfered with detection of the visual target. In addition, the target-colored distractor also lead to the corresponding attentional set for color entering the focus of attention, further restricting the capacity to process the auditory stimulus as its features were associated with a different attentional set.

Although our results are consistent with the notion that contingent capture involves an amodal bottleneck in neural processing, it could be argued that the target-colored distractor may have primed the hand that was used to respond to visual stimuli. For auditory targets such priming could have led to longer reaction times because the other hand was required to make a response. Importantly, because the distractor was not predictive of the target’s modality or identity, such an effect could not have arisen due to participants intentionally using the distractor as a cue. Indeed, the distractor-to-target delay was likely too short to allow for intentional response preparation (Adam et al., 2005). Thus, any response priming was automatic and was also contingent upon attentional set, as it was specific to the target-colored distractor. In this context it should be noted that although participants searched for a colored item, their task was to report the identity of that item. Discrimination judgments of this sort, which are orthogonal to the dimension along which the cue varies, typically avoid response priming (for a review of cross-modal effects, see Spence et al., 2004). Thus, it seems unlikely that the target-colored distractor simply primed the relevant hand. It remains possible, however, that the target-colored distractor cued the stimulus-response mapping for visual targets (e.g., Mattler, 2006; Reuss et al., 2011), which could induce a cost when a switch to the auditory attention set (and its stimulus-response mapping) was required (Du et al., 2014). This argument is similar to our contention that the target-colored distractor brought into focus an attentional set for color, but emphasizes more the response-selection rather than discrimination stage of processing. Future research will be needed to determine the extent to which visual contingent capture affects the perception, decision, and response stages of sound detection.

In summary, we have shown that contingent capture by an irrelevant visual stimulus that matches top-down attentional control settings interferes with detection of both visual and auditory targets. The current results are consistent with the hypothesis that, in addition to a shift in spatial attention, contingent capture involves a serial stage of neural processing that is limited in capacity. More generally, the results are consistent with the notion that attentional selection acts to prioritize and restrict access to this capacity-limited processing stage.

## AUTHOR CONTRIBUTIONS

Conceived and designed the experiments: Marc R. Kamke, Jill Harris. Acquired the data: Jill Harris. Analyzed the data: Marc R. Kamke, Jill Harris. Wrote and/or revised the paper: Marc R. Kamke, Jill Harris.

## Conflict of Interest Statement

The authors declare that the research was conducted in the absence of any commercial or financial relationships that could be construed as a potential conflict of interest.
